# Expression of the hypermethylated in cancer gene (*HIC-1*) is associated with good outcome in human breast cancer

**DOI:** 10.1054/bjoc.2001.2163

**Published:** 2001-12

**Authors:** G Nicoll, D N Crichton, H E McDowell, N Kernohan, T R Hupp, A M Thompson

**Affiliations:** Department of Surgery and Molecular Oncology, Department of Molecular and Cellular Pathology, University of Dundee, Dundee, DD1 9SY, UK; Department of Medical Sciences Gastroenterology Laboratory, University of Edinburgh, EH4 2XU, UK

**Keywords:** *HIC-1*, hypermethylation, *p53*, prognosis, breast cancer

## Abstract

A new cancer gene, *HIC-1* (Hypermethylated in Cancer) telomeric to *p53* on chromosome 17p may be of clinical importance in sporadic breast cancer. Regional DNA hypermethylation of 17p13.3 resulting in suppression of gene expression has been shown to precede 17p structural changes in human carcinogenesis. In addition, loss of heterozygosity studies have suggested clinically significant involvement of a gene on 17p13.3 associated with poor prognosis in breast cancer. Using RT-PCR analysis, we demonstrate that the MCF7 (wild type p53) cell line expressed HIC-1 transcripts but the MDAMB231 (mutant p53) cell line did not, suggesting loss of *HIC-1* expression and *p53* malfunction may be synergistic events in sporadic breast cancer. HIC-1 expression was examined using RT-PCR on RNA extracted from 50 primary untreated, human breast cancers and was detected in only 7/50 (14%) cancers. All seven patients with HIC-1 expression were alive without disease recurrence after 8 years follow-up and 5/7 had detectable p53 wild type mRNA expression. This suggests that retained HIC-1 expression may offer a survival advantage. However the seven cancers had 17p13.3 loss of heterozygosity (LOH; four patients), a feature previously associated with poor prognosis, or were homozygous (three patients) suggesting there may be two genes at 17p13.3 involved in breast carcinogenesis. Using a demethylating drug 5-aza-2′-deoxycytidine (DeoxyC), HIC-1 expression was restored in the MDAMB231 cells, also suggesting restoration of HIC-1 function by reversing *HIC-1* hypermethylation may offer a therapeutic avenue in breast cancer. © 2001 Cancer Research Campaign http://www.bjcancer.com


*British Journal of Cancer* (2001) **85**(12), 1878–1882
© 2001 Cancer Research Campaign
doi: 10.1054/ bjoc.2001.2163, available online at http://www.idealibrary.com on IDEAL

http://www.bjcancer.com

### Expression of the hypermethylated in cancer gene (*HIC-1*) is associated with good outcome in human breast cancer

**G Nicoll[1], DN Crichton[2], HE McDowell[1], N Kernohan[3], TR Hupp[3] and AM Thompson[1]**

[1]Department of Surgery and Molecular Oncology; and [3]Department of Molecular and Cellular Pathology, University of Dundee, Dundee DD1 9SY, UK;
[2]Department of Medical Sciences Gastroenterology Laboratory, University of Edinburgh EH4 2XU, UK

**Summary** A new cancer gene, *HIC-1* (Hypermethylated in Cancer) telomeric to *p53* on chromosome 17p may be of clinical importance in sporadic breast cancer. Regional DNA hypermethylation of 17p13.3 resulting in suppression of gene expression has been shown to precede 17p structural changes in human carcinogenesis. In addition, loss of heterozygosity studies have suggested clinically significant involvement of a gene on 17p13.3 associated with poor prognosis in breast cancer. Using RT-PCR analysis, we demonstrate that the MCF7 (wild type p53) cell line expressed HIC-1 transcripts but the MDAMB231 (mutant p53) cell line did not, suggesting loss of *HIC-1* expression and *p53* malfunction may be synergistic events in sporadic breast cancer. HIC-1 expression was examined using RT-PCR on RNA extracted from 50 primary untreated, human breast cancers and was detected in only 7/50 (14%) cancers. All seven patients with HIC-1 expression were alive without disease recurrence after 8 years follow-up and 5/7 had detectable p53 wild type mRNA expression. This suggests that retained HIC-1 expression may offer a survival advantage. However the seven cancers had 17p13.3 loss of heterozygosity (LOH; four patients), a feature previously associated with poor prognosis, or were homozygous (three patients) suggesting there may be two genes at 17p13.3 involved in breast carcinogenesis. Using a demethylating drug 5-aza-2′-deoxycytidine (DeoxyC), HIC-1 expression was restored in the MDAMB231 cells, also suggesting restoration of HIC-1 function by reversing *HIC-1* hypermethylation may offer a therapeutic avenue in breast cancer. © 2001 Cancer Research Campaign http://www.bjcancer.com

**Keywords**: *HIC-1*; hypermethylation; *p53*; prognosis; breast cancer

**Abbreviations**: *HIC-1*: hypermethylated in cancer gene 1; DNA: Deoxyribonucleic acid; mRNA: messenger ribonucleic acid; RT-PCR: reverse transcription-polymerase chain reaction; LOH: loss of heterozygosity; DeoxyC: 5-aza-2′-deoxycytidine.

Worldwide there are one million women newly diagnosed with breast cancer each year. Despite advances in early detection through screening and family history clinics, chemotherapy and endocrine therapy, breast cancer remains a common cause of death with survival figures of 75% at 5 years and 60% at 10 years. While the underlying genetic events in familial breast cancer (which accounts for 5–10% of women with breast cancer) are attributed to mutations in *BRCA1*, *BRCA2*, the ataxia telangiectasia gene or the *p53* gene, the genetic events for the 90% of breast cancer which is sporadic are less clear.

In sporadic breast cancer, allele losses (which can reveal the location of tumour suppressor genes) from the short arm of chromosome 17 are amongst the most frequent genetic alterations observed. At least two regions on 17p have been identified in loss of heterozygosity (LOH) or allelic imbalance studies of paired blood and breast cancer DNA (Stack et al, 1995). One of these regions, the *p53* gene at 17p13.1, has attracted wide attention as a tumour suppressor gene, transcription factor and mediator of apoptosis in many types of cancer (Steele et al, 1999), including breast cancer (Ziyaie et al, 2000). A second region, defined by markers YNZ22 (D17S5) and 144D6 (D17S30), is situated in band 17p13.3, 20 Mb telomeric to the *p53* tumour suppressor gene, and may contain more than one gene involved in carcinogenesis.

High frequencies (40–75%) of LOH (Coles et al, 1990; Thompson et al, 1990, Stack et al, 1995) and recent international collaborative data, including that from our group (Phelan et al, 1998), confirm the existence and clinical relevance of a tumour suppressor gene that is distinct from *p53* at 17p13.1 (Coles et al, 1990) and also displays allelic loss in a number of other human malignancies. LOH at YNZ22 (17p13.3) appears to be an early event in breast carcinogenesis since it is observed in ductal carcinoma in situ (Aldaz et al, 1995) and, in ovarian cancer, precedes *p53* deletion (Phillips et al, 1996). More detailed studies of allelic imbalance in breast cancer, using markers spanning 17p13.3 has further refined the deletion map (Stack et al, 1995). Furthermore, LOH at YNZ22 can occur in the absence of *p53* mutation, deletion or over-expression (Cornelis et al, 1994; Thompson et al, 1998) suggesting both regions may be of clinical importance in sporadic breast cancer (Thompson et al, 1998; Liscia et al, 1999). In addition, supportive evidence for the involvement of a tumour suppressor gene, distinct from *p53* on chromosome 17p, is strengthened by the functional suppression of malignancy observed following the transfection of chromosome 17 (but not with chromosome 13) into breast cancer cell lines by micro cell-mediated chromosome transfer and analysis of the resulting deletions (Casey et al, 1993; Theile et al, 1995). LOH at 17p13.3 is

*Received 6 June 2001*
*Revised 9 August 2001*
*Accepted 19 September 2001*

*Correspondence to:* AM Thompson also associated with an aggressive phenotype being significantly correlated with disease recurrence and death due to breast cancer (Thompson et al, 1998; Nagai et al, 1995).

Recently, a candidate tumour suppressor gene *HIC-1* (hypermethylated in cancer) has been described on chromosome 17p13.3 (Makos-Wales et al, 1995) in the region with a high level of allelic loss in sporadic breast cancer (Phelan et al, 1998). *HIC-1* was identified because of its association with a CpG-rich region or 'CpG island'. These islands are short and dispersed regions of DNA with a high frequency of CpG dinucleotides relative to the genome generally. A minor fraction (about 2%) is associated with the 5′-ends and hence the promoters and transcription start sites of all housekeeping genes and of some tissue specific genes and have thus been postulated to be sites of interaction between transcription factors and promoters (Cross et al, 1994). The methylation state of the base cytosine modulates the overall genomic pattern of chromatin organization and gene expression and the CpG dinucleotide is the principal, but not exclusive, site of DNA methylation.

Methylation of DNA can lead to mutation through the conversion of methylated cytosine to thymidine by deamination. Hypermethylation of normally unmethylated CpG islands has been implicated as one alternative mechanism to chromosomal deletion for the functional inactivation of tumour suppressor genes. The *HIC-1* gene, which is entirely encompassed within a CpG island, is aberrantly hypermethylated and transcriptionally inactivated in several types of human cancers including breast cancer (Fujii et al, 1998) and may play a critical role in mammalian development (Carter et al, 2000). Analysis of the putative primary protein sequence suggests the gene product has five Krupple-type $Cys_2$-$His_2$ zinc-fingers and an N-terminal protein interaction domain characteristic of a subset of zinc-finger transcription factors (Deltour et al, 1998; Guerardel et al, 1999). Nucleotide sequence analysis has revealed a consensus p53 binding site in the 5′ flanking region 4 kb upstream from the transcription start site and *HIC-1* is postulated to be a potential downstream target of *p53*. Functional studies have demonstrated activation of HIC-1 transcription by exogenous *p53* in a colon cancer cell line and transfection of the *HIC-1* gene into cultured colon cancer cells was found to suppress cell growth (Makos-Wales, 1995).

Thus, while mutation or LOH of tumour suppressor genes are common events in breast cancer, silenced gene transcription associated with hypermethylation is an alternate mechanism to chromosomal deletion for the inactivation of tumour suppressor genes. The aim of the present study was to determine the frequency of *HIC-1* transcription in a cohort of 50 breast cancers and to compare this event with data on 17p allelic loss, p53 expression and clinical/pathological variables including disease outcome for the same cancers.

### MATERIALS AND METHODS

Samples of primary breast cancer tissue were collected from 50 female Caucasian patients with a histological diagnosis of invasive carcinoma of the breast. All 50 patients were clinically stage T1-T3, N0-N1 and M0, previously untreated and underwent mastectomy or wide local excision of the cancer. Histological type, tumour size and nodal involvement were recorded prospectively on the basis of pathology findings; tumour grading was not performed. Tumour samples were handled identically and snap frozen in liquid nitrogen prior to storage at –80˚C until analysis. Following accrual of laboratory data, statistically significant associations were sought between HIC-1 expression and clinical/pathological variables using a two-tailed Fisher's exact test.

### Extraction of RNA

RNA was extracted as previously described (Thompson et al, 1990). Briefly, 500 mg frozen tissue was pulverized and disrupted in 3M lithium acetate/6M urea (2 ml/100 mg tissue) and incubated at 4˚C overnight. DNA was sheared using a Soniprep 150 ultrasonic disintegrator (MSE Scientific Instruments, Crawley, UK) and the RNA recovered by centrifugation at 12000 rpm. The pellet was resuspended in 6 ml 10 mM Tris.HCl buffer pH7 containing 0.1% sodium dodecyl sulphate, 50 μg/ml of proteinase K (Boehringer, Mannheim, FRG) and incubated at 37˚C for 20 min. Protein was extracted with phenol equilibrated with 0.1 M Tris.HCl pH7 followed by chloroform:isoamyl alcohol (24:1). Following ethanol precipitation at –20˚C the RNA was recovered by centrifugation, dissolved in RNase-free distilled water and stored in aliquots at –70˚C. The quantity and purity of the RNA was assessed by spectrophotometry.

In addition, RNA from the human breast cancer cell lines MCF7 and MDAMB231 was also examined in this study. The cells were grown at 37˚C in a humidified atmosphere containing 5% $CO_2$, in Dulbecco's modified Eagle's medium (Life Technologies, Paisley, UK) containing 2 mM glutamine, 0.1 mM non-essential amino acids, 10% fetal calf serum, 1000 U/ml penicillin and 100 μg/ml streptomycin. Subconfluent cultures were harvested by aspiration, the cells washed with phosphate buffered saline and RNA isolated with an RNA extraction kit (Qiagen, Crawley, UK) according to the manufacturer's instructions.

### RT-PCR

Aliquots of RNA were digested with 10 U/ug RNase-free DNase (Boehringer) for 1 h at 37˚C to remove contaminating genomic DNA. An equal amount of each RNA sample was also incubated under identical conditions in the absence of the enzyme. Both enzyme-treated and untreated samples were purified with an RNA purification kit (Qiagen) according to the manufacturer's instructions.

1–5 μg of the RNA was reverse transcribed using oligo$(dt)_{15}$ with superscript II reverse transcriptase (Life Technologies) according to the manufacturer's instructions. HIC-1 transcripts were amplified in a Hybaid Touchdown thermal cycler from the cDNA in 50 μl AmpliTaq Gold buffer volumes containing 10% DMSO, 1.25U AmpliTaq Gold DNA polymerase (PE Biosystems), 200 μm of each dNTP, 2.5 mM $MgCl_2$ and l μM of each primer. Primer sequences for *HIC-1* (derived from Genbank Accession no. L41919) (Table 1) were designed to amplify a 242bp fragment from exon 2 of the *HIC-1* gene. To activate the AmpliTaq Gold DNA polymerase the reaction mixture was incubated at 95˚C for 12 min. Amplification conditions were 35 cycles of 95˚C for 1 min (denaturation) and 72˚C for 1 min (annealing and extension). PCR was also performed using primers specific for *MDM2* and *β-Actin* as positive control reactions for successful cDNA synthesis (Table 1). MDM2 transcripts were amplified in a final volume of 50 μl AmpliTaq buffer containing 1.25U AmpliTaq (PE Biosystems), 200 μM of each dNTP, 1.5 mM $MgCl_2$, and 1 μM of each primer. Amplification conditions were 95˚C for 5 min (initial denaturation) followed by 35 cycles of 95˚C for 1 min (denaturation), 65˚C for

© 2001 Cancer Research Campaign 

**Table 1** Primer sets for *HIC-1*, *MDM2* and β actin expression studies by RT-PCR

| Primer set | Sense primer | Antisense primer | Size bp |
|---|---|---|---|
| *HIC-1* | ATGGTGAGCCCGGCCGTGTTCC | TAGCCGCCGCCGCCGCCGCCGC | 242 |
| *MDM*$_2$ | GGGAGATATGTTGTGAAA | AGATTCAACTTCAAATTC | 152 |
| β actin | ATCTGGCACCACACCTTCTACA ATGAGCTGCG | CGTCATACTCCTGCTTGCTGATCC ACATCTGC | 838 |

1 min (annealing) and 72˚C for 1 min (extension). β-Actin transcripts were produced using RT-PCR control amplimers (Clontech) according to the manufacturer's instructions. Following amplification, 5 µl of DNA loading buffer (bromophenol blue in 50% w/v glycerol) was added to each PCR reaction, PCR products were resolved on 1% agarose gels, containing 0.5 µg/ml ethidium bromide and visualized under UV illumination. A 1kb DNA ladder was used as size standards. PCR positive controls included the amplification of *HIC-1* from genomic tonsil DNA, *MDM2* from plasmid CMX-mdm2 containing full length human *MDM2* cDNA (gifted from Christine Blattner) and human β actin control (Clontech). Negative controls consisted of all the components of the PCR excluding target sequences.

### p53 expression and 17p13.3 allele loss (LOH)

From the same samples used for RT-PCR studies, total RNA for the 50 breast cancers was used for northern blots and p53 mRNA expression detected using the 2.1 kb cDNA clone php53Bam, the filters stripped then reprobed for alpha actin as internal control as previously described (Thompson et al, 1990). DNA was extracted from cancer tissue immediately adjacent to that used for the RNA studies and from the patient's venous blood lymphocytes (Thompson et al, 1990). Lymphocytes were used in preference to apparently normal breast tissue given the adverse cosmetic impact excising further breast tissue could have on breast conservation and the possibility of additional foci of cancer in mastectomy specimens. Allele loss (using a cut-off of 50% band strength) was detected using blood/tumour pairs for the 50 patients using Southern blots probed with YNZ22 which maps to 17p13.3 as previously described (Thompson et al, 1990).

### Reactivation of HIC-1 expression in MDAMB231

MDAMB231 cells were seeded at 1 × 10$^5$ per 75-mm$^2$ flask in phenol red free Minimum Essential Medium (GibcoBRL) containing 2 mM glutamine, 10% fetal calf serum, 1000 U/ml penicillin and 100 µg/ml streptomycin. After 24 h the cells were incubated with freshly prepared medium containing 3 × 10$^{-7}$M 5-aza-2′-deoxycytidine (DeoxyC); (Sigma). DeoxyC-containing medium was replaced on days 5 and 8. The cells were harvested on day 10 for RNA extraction and RT-PCR as described above.

### RESULTS

HIC-1 expression was detected in the MCF7 cell line and in 7/50 breast cancers (14%) by RT-PCR, but despite positive actin and MDM2 controls, no expression was detected in the MDAMB231 cell line nor in 43/50 (86%) of the cancers (Figure 1). The HIC-1 RT-PCR product was sequenced and the published *HIC-1* sequence confirmed (data not shown). In the MDAMB231 cell line, consititutional expression of beta actin and mdm2 genes was detected (Figure 2A) indicating the successful transcription of

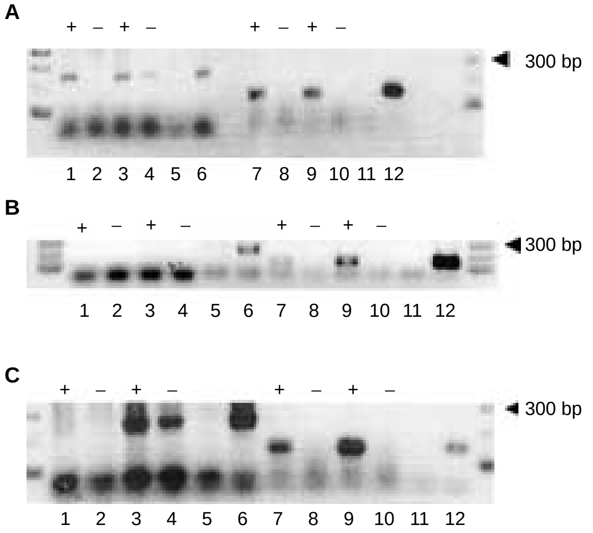

**Figure 1** HIC-1 expression in breast cancer cell lines MCF7, MDAMB231 and a human breast cancer. RT-PCR was used to detect HIC-1 mRNA expression (242 bp) and MDM2 expression (152 bp) in: (A) MCF7. (B) MDA-MB-231. (C) Breast cancer (patient number 38). +/– indicate whether reverse transcriptase (RT) was added to (+) or omitted from (–) the reaction. Lane 1–6 HIC-1 RT-PCR, Lane 7–12 MDM2 RT-PCR. Lane 1: DNase treated. Lane 2: DNase treated no RT. Lane 3: no DNase. Lane 4: no DNase, no RT. Lane 5: negative control for PCR reaction. Lane 6: positive control. Lane 7: DNase treated. Lane 8: DNase, no RT. Lne 9: no DNase. Lane 10: no DNase, no RT. Lane 11: negative control for PCR reaction. Lane 12: positive control.

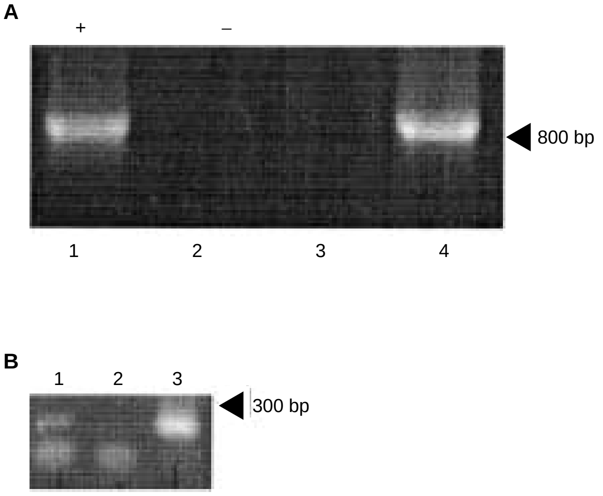

**Figure 2** RT-PCR was used to detect actin mRNA (838 bp) expression in MDAMB231 (Figure 2A) and HIC-1 mRNA expression in DeoxyC treated MDAMB231 (Figure 2B): **A** Beta actin expression in MDAMB231. Lane 1: DNase treated. Lane 2: DNase treated no RT. Lane 3: negative control for PCR reaction. Lane 4: positive control. +/– indicate whether reverse transcriptase was added to (+) or omitted from (–) the reaction.
**B** Restoration of HIC-1 expression in MDAMB231. Lane 1: DNase treated; 5-aza-2′-deoxycytidine treated-MDAMB231: Lane 2: negative control for PCR reaction. Lane 3: positive control

 *© 2001 Cancer Research Campaign*

**Table 2** **A** HIC-1 expression, p53 expression and **B** YNZ 22 allele loss

| A | | p53mRNA expression | |
|---|---|---|---|
| | | Yes | No |
| HIC-1 expression | yes | 5 | 2 |
| HIC-1 expression | no | 25 | 18 |

| B | | YNZ22 | | |
|---|---|---|---|---|
| | | Allele loss | No allele loss | Homozygous |
| HIC-1 expression | yes | 4 | 0 | 3 |
| HIC-1 expression | no | 17 | 13 | 13 |

cDNA. Furthermore, HIC-1 expression was restored following treatment with the demethylating drug DeoxyC (Figure 2B). p53 mRNA expression was detected in 32/50 (64%) cancers, YNZ22 loss of heterozygosity (LOH) demonstrated in 21/50 (42%) with 16/50 (32%) patients constitutionally homozygous (Thompson et al, 1998).

Among the seven cancers where HIC-1 expression was detected using RT-PCR, 5/7 (71%) had detectable p53 expression which was wild type, (Thompson et al, 1992) (Table 2A) and 4/7 had YNZ22 LOH (3 were homozygous) (Table 2B). For the 43/50 cancers which tested negative for HIC-1 expression, 25/43 were positive for p53 expression and 17/43 (34%) had YNZ22 LOH (13 no loss, 13 homozygous).

All seven patients in whom cancer HIC-1 expression was detected were alive after 8 years follow-up (Table 3); 3/7 were oestrogen receptor (ER) positive and 1/7 was histologically node positive. For the remaining 43 patients in whom HIC-1 expression could not be detected, 28/43 (65%) were ER positive, 23/43 (54%) were node positive and 23/43 (54%) were alive without disease recurrence after 8 years follow-up (Table 3).

HIC-1 expression (detected in seven cancers) was associated with YNZ22 LOH ($P$ = 0.015, two-tailed Fisher's exact test), absence of node metastasis at diagnosis ($P$ = 0.045) and all seven patients were alive without recurrence at 8 years follow-up ($P$ = 0.04). There was no significant association between HIC-1 expression and oestrogen receptor status, histological tumour size or histological type (all the cancers studied were ductal carcinoma of no special type).

### DISCUSSION

Following the demonstration in this study (using reverse transcription polymerase chain reaction, RT-PCR) that the hormone-responsive cell line MCF7 expressed HIC-1 transcripts and wild-type p53 but that the hormone receptor-negative, MDA-MB-231 cell line which expresses mutant p53 did not transcribe *HIC-1*, we have examined a pilot series of 50 sporadic breast cancers for *HIC-1* transcription and compared this with p53 mRNA expression, YNZ22 allele loss, node status, oestrogen receptor expression and disease recurrence. In addition, we have demonstrated that HIC-1 expression can be restored by the use of a demethylating drug, DeoxyC, in a breast cancer cell line without detectable HIC-1 expression prior to treatment.

The MCF7 HIC-1 expression data contrasts with the initial study of *HIC-1* and breast cancer which failed to identify HIC-1 expression in MCF7 by the RNase protection assay (Makos-Wales et al, 1995); however, in keeping with our current report, subsequent studies have suggested HIC-1 is expressed in MCF7 (Fujii et al, 1998).

HIC-1 transcripts were demonstrated in only 7/50 (14%) cancers despite positive internal controls for MDM2 and actin demonstrating successful RT-PCR from the RNA of all 50 cancers. This concords with the only published series of 39 breast cancers (Fujii et al, 1998) where, using an alternative experimental approach, HIC-1 appeared to be inactive in 67% of cancers.

For the first time we have identified an association between retained HIC1 expression and absence of node metastasis at diagnosis ($P$ = 0.045); indeed, all seven patients were alive without recurrence at 8 years follow-up ($P$ = 0.04). In the present series there was no significant association between HIC-1 expression and oestrogen receptor status (there is no clinical data cited by Fujii et al, 1998). In the present study we have also shown that HIC-1 expression (seven cancers) was associated with YNZ22 LOH ($P$ = 0.015, two-tailed Fisher's exact test) a result which concurs with that previously reported (Fujii et al, 1998). Given the link between YNZ22 LOH and poor prognosis in breast cancer (Thompson et al, 1998; Liscia et al, 1999) one might expect HIC-1 expression would correlate with poor prognosis. These differing associations suggest that *HIC-1* may be distinct from a second gene involved in breast cancer marked by YNZ22. In addition *HIC-1* may protect against the deleterious effects of YNZ22 LOH. Moreover, in our series, HIC-1 expression was not associated with p53 expression or with *p53* mutation (Thompson et al, 1998) although activation of HIC-1 expression by *p53* has been reported in colorectal cancer cell lines (Makos-Wales et al, 1995) and *p53* mutation has been linked to *HIC-1* methylation in four cancers (Fujii et al, 1998).

Regional DNA hypermethylation at 17p13.3 has been shown to precede 17p structural changes and is thus an early event in the progression of renal tumours (Makos et al, 1993) but, conversely, is a late event in haemopoietic malignancies (Issa et al, 1997). In breast cancer the hypermethylation of HIC-1 has been demonstrated in cultured neoplastic cells (Makos-Wales et al, 1995) although other mechanisms of *HIC-1* inactivation such as mutation may occur.

The failure to detect *HIC-1* transcripts in MCF7 cells in an earlier study (Makos Wales et al, 1995) may be attributable to their use of a less sensitive assay (RNase protection) or a consequence of phenotypic divergence of the cell lines maintained under separate culture conditions in different laboratories. It has been reported that one *HIC-1* allele is methylated in normal breast epithelium and that such hemimethylation predisposes to increased risk of breast cancer through mutation of the other allele (Fujii et al, 1998). In contrast, methylation of the *HIC-1* CpG

**Table 3** HIC-1 expression and clinical/pathological parameters

| | | ER positive | ER negative | Node positive | Node negative | Disease recurrence |
|---|---|---|---|---|---|---|
| HIC-1 | Yes ($n$ = 7) | 3 | 4 | 1 | 6 | 0 |
| Expression | No ($n$ = 43) | 28 | 15 | 23 | 20 | 20 |

© 2001 Cancer Research Campaign island extends to a sequence between the intron 2 and exon 3 junction in normal haemopoietic cells but occurs beyond this boundary in newly diagnosed acute myeloid leukaemias (Melki et al, 1999). Thus the extent of methylation, as opposed to methylation per se may be a more reliable indicator of gene inactivation. In the present study we have investigated breast cancer RNA directly by RT-PCR for evidence of HIC1 transcription.

Promoter-associated CpG islands can be chemically modified by methylation of cytosine that prevents expression of the gene. This could be of particular relevance in breast cancer as oestrogen receptor genes may also become hypermethylated (Issa et al, 1997; Li et al, 1998), a potential mechanism for resistance of oestrogen receptor positive breast cancers to endocrine therapy, and gene methylation of the oesotrogen receptor increases with age (Li et al, 1998), as does breast cancer. Thus, altering the methylation status of cancer cells through pharmacological inhibition of DNA methyltransferase with resultant DNA demethylation (Issa et al, 1997) offers a novel therapeutic approach to reactivating tumour suppressor function of genes such as *HIC-1*, as we have demonstrated here with MDAMB231, but may also be relevant to reactivating the function of what are currently therapeutically useful genes such as oestrogen receptor. Indeed, the demethylating effects on gene expression (and hence putative reactivation) by the drug 5 aza-2′-deoxycytidine are undergoing further study in our laboratories. Immunohistochemical analysis of 5 aza-2′- deoxycytidine treated MDAMB231 cells has demonstrated an increased expression of p53, the proto-oncogene erbB2, and the oestrogen responsive protein pS2 as well as re-expression of HIC-1 (data not shown).

Thus, *HIC-1* is a prime candidate gene in the development of sporadic breast cancer since it is located in a chromosomal region of 17p distinct from *p53* that frequently undergoes LOH. However, the relative contributions of mutation, deletion and methylation leading to *HIC-1* inactivation in breast cancer remain unclear. In addition, the putative role for *HIC-1* as a tumour suppressor gene, the relationship to *p53* and the clinical importance of *HIC-1* (with potential for therapeutic attack) in breast cancer require further elucidation. Our data suggest that events on chromosome 17p13 including *HIC-1* and *p53* may be of more interest than hitherto expected.

### ACKNOWLEDGEMENTS

We thank Christine Blattner for the human wild type *MDM2* plasmid, CMX-Mdm2 and Mark Saville for constructive criticisms of the manuscript. This work is supported by Breast Cancer Research (Scotland), the Chief Scientists Office and the MRC/CRC Tayside tissue/tumour bank; we thank them for their support.

### REFERENCES

Aldaz CM, Chen T, Sahin A, Cunningham J and Bondy M (1995) Comparitive allelotype of in situ and invasive human breast cancer: high frequency of microsatellite instability in lobular breast carcinomas. *Cancer Res* **55**: 3976–3981

Carter MG, Johns MA, Zeng X, Zhou L, Zink MC, Mankowski JL, Donovan DM and Baylin SB (2000) Mice deficient in the candidate tumor suppressor gene *Hicl* exhibit developmental defects of structures affected in the Miller-Dieker syndrome. *Hum Mol Genet* **9**: 413–419

Casey G, Plummer S, Hoeltge G, Scanlon D, Fasching C and Stanbridge EJ (1993) Functional evidence for a breast cancer suppresor gene on shromosome 17. *Hum Mol Genet* **2**: 1921–1927

Coles C, Thompson AM, Elder PA, Cohen BB, MacKenzie IM, Cranston G, Chetty U, Mackay J, Macdonald M, Nakamura Y, Hoyheim B and Steele CM (1990) Evidence implicating at least two genes on chromosome 17 p in breast carcinogenesis. *Lancet* **336**: 761–763

Cornelis RS, van Vliet M, Vos CB, Cleton-Jansen AM, van de Vijver MJ, Peterse JL, Khan PM, Borresen AL, Cornelisse CJ and Devilee P (1994) Evidence for a gene on 17 p13.3, distal to TP53, as a target for allele loss in breast tumors without p53 mutations. *Cancer Res* **54**: 4200–4206

Cross SH, Charlton JA, Nan X and Bird AP (1994) Purification of CpG islands using a methylated DNA binding column. *Nature* **6**: 236–244

Deltour S, Guerardel C, Stehelin D and Leprince D (1993) the carboxy-terminal end of the candidate tumor suppressor gene HIC-1 is phylogenetically conserved. *Biochim Biophys Acta* **1443**: 230–232

Fujii H, Biel M, Zhou W, Weitzman S, Baylin SB and Gabrielson E (1998) Methylation of the HIC-1 candidate tumor suppressor gene in human breast cancer. *Oncogene* **16**: 2159–2164

Guerardel C, Deltour S and Leprince D (1999) Evolutionary divergence in the broad complex tramtrak and bric a brac/poxviruses and zinc finger domain from the candidate tumor suppressor gene hypermethylated in cancer. *FEBS Lett* **451**: 253–256

Issa JP, Baylin SB and Herman JG (1997) DNA methylation changes in hematologic malignancies: biologic and clinical implications. *Leukemia* **11** (Suppl) 1 S7–S11

Li Q, Jedlicka A, Ahuja N, Gibbons MC, Baylin SB, Burger PC and Issa JP (1998) Concordant methylation of the ER and N33 genes in glioblastoma multiforme. *Oncogene* **16**: 3197–3202

Liscia D, Morizio R, Venesio T, Palenzona C, Donadio M and Callahan R (1999) Prognostic significance of loss of heterozygosity at loci on chromosome 17 p13.3-ter in sporadic breast cancer is evidence for a putative tumour suppressor gene. *Br J Cancer* **80**: 821–826

Makos M, Nelkin BD, Chazin VR, Cavenee WK, Brodeur GM and Baylin SB (1993) Regional DNA hypermethylation at D17S5 precedes 17 p structural changes in the progression of renal tumors. *Cancer Res* **53**: 2719–2722

Makos-Wales M, Biel MA, el Deiry W, Nelkin BD, Issa J-P, Cavanee WK, Kuerbitz SJ and Baylin SB (1995) P53 activates expression of HIC-1, a new candidate tumour suppressor gene on 17 p13.3. *Nature Medicine* **1**: 570–577

Melki JR, Vincent PC and Clark SJ (1993) Cancer-specific region of hypermethylation identified within the HIC-1 putative tumour suppressor gene in acute myeloid leukaemia. *Leukaemia* **6**: 877–883

Nagai MA, Medeiros AC, Brentani MM, Marques LA, Mazoyer S and Mulligan LM (1995) Five distinct deleted regions on chromosome 17 defining different subsets of human primary breast tumours. *Oncolgy* **52**: 448–453

O'Neill M, Campbell SJ, Save V, Thompson AM and Hall PA (1998) An immunochemical analysis of mdm2 expression in human breast cancer and the identification of a growth-regulated cross-reacting species p170. *J Pathol* **186**: 254–261

Phelan CM, Borg A, Cuny M, Crichton DN and 22 others (1998) Consortium study on 1280 breast carcinomas: allelic loss on chromosome 17 targets subregions associated with family history and clinical parameters. *Cancer Res* **58**: 1004–1012

Phillips NJ, Ziegler MR, Radford DM, Fair KL, Steinbeck T, Xynos FP and Donis-Keller H (1996) Allelic deletion on chromosome 17p13.3 in early ovarian cancer. *Cancer Res* **56**: 606–611

Stack M, Jones D, White G, Liscia D, Venesio T, Casey G, Crichton D, Varley J, Mitchell E, Heighway J and Santibanez-Koref M (1995) Detailed mapping and loss of heterozygosity analysis suggests a suppressor locus involved in sporadic breast cancer within the distal region of chromosome band 17p13.3. *Human Molecular Genetics* **4**: 2047–2055

Steele RJC, Thompson AM, Hall PA and Lane DP (1998) The p53 tumour suppressor gene. *Br J Surg* **85**: 1460–1467

Theile M, Hartmann S, Scherthan H, Arnold W, Deppert W, Frege R, Glabb F, Haensch W and Scherneck S (1995) Suppression of tumorigenicity of breast cancer cells by transfer of human chromosome 17 does not require transferred BRCA1 and p53 genes. *Oncogene* **10**: 439–447

Thompson AM, Steel CM, Chetty U, Hawkins RA, Miller WR, Carter DC, Forrest APM and Evans H (1990) p53 gene mRNA expression and chromosome 17p allele loss in breast cancer. *Br J Cancer* **61**: 74–78

Thompson AM, Anderson TJ, Condie A, Prosser J, Chetty U, Carter DC, Evans HJ and Steel CM (1992) P53 allele losses, mutations and expression in breast cancer and their relationship to clinico-pathological parameters. *Int J Cancer* **50**: 528–532

Thompson AM, Crichton DN, Elton RA, Clay MF, Chetty U and Steel CM (1998) Allelic imbalance at chromosome 17p13.3 (YNZ22) in breast cancer is independent of p53 mutation or p53 overexpression and is associated with poor prognosis at medium term follow up. *Br J Cancer* **77**: 797–800

Ziyaie D, Hupp TR and Thompson AM (2000) p53 and breast cancer. *The Breast* **9**: 239–246

 *© 2001 Cancer Research Campaign*
